# Microwave Puffing—Mediated Structural Modification of Soy Protein: Effects on Dynamic Changes in Key Properties During Soybean Paste Fermentation and Umami Improvement

**DOI:** 10.3390/foods15142542

**Published:** 2026-07-18

**Authors:** Jialu Tong, Guanlong Li, Xiaolan Liu, Xiqun Zheng

**Affiliations:** 1 College of Food Science, Heilongjiang Bayi Agricultural University, Daqing 163319, China; tongjialu9518@163.com; 2 College of Food and Bioengineering, Qiqihar University, Qiqihar 161006, China; 03580@qqhru.edu.cn

**Keywords:** microwave, protein structure, soybean paste, umami

## Abstract

This study investigated the effects of microwave puffing-induced soy protein structural modification on the dynamic changes in physicochemical properties and umami formation during soybean paste fermentation. The results indicated that optimal microwave puffing (400 W, 90 s) disrupts protein non-covalent bonds, depolymerizes protein aggregates, exposes the internal hydrophobic groups to enhance surface hydrophobicity, forms a loose porous structure, and creates favorable conditions for enzymatic hydrolysis during soybean paste fermentation. Conversely, excessive treatment (400 W, >90 s) induced protein re-aggregation and inhibited proteolysis, as confirmed by SEM. Furthermore, microwave puffing (400 W, 90 s) promoted *Aspergillus oryzae* growth and protease activity, and significantly improved physicochemical properties of soybean paste, including lower pH and higher contents of total acid (5.8%), amino acid nitrogen (11.8%), small peptides (13.3%), and reducing sugars (14.95%) (*p* < 0.05), plus a brighter, redder color. LC-MS/MS revealed microwave puffing (400 W, 90 s) reshaped the peptide profile of soybean paste, increasing the relative abundance of the umami amino acids (Glu, Asp) and sweet amino acid (Ala) and decreasing proportions of the bitter amino acids in the peptides. Sensory and electronic tongue analyses confirmed enhanced umami and weaker bitterness and saltiness. These findings demonstrate that microwave puffing (400 W, 90 s) effectively improves the quality and flavor characteristics of soybean paste, providing technical support for high-quality fermented soybean products.

## 1. Introduction

As a quintessential fermented soybean product, soybean paste stands among China’s oldest and most traditional seasonings [1]. Renowned for its rich savory flavor, distinctive taste profile, and nutritional value, it is widely used and highly regarded in many regions worldwide [2]. The quality and market competitiveness of soybean paste hinge primarily on its flavor characteristics, among which umami is the core sensory attribute that determines consumer preference. It mainly comes from umami substances produced during the fermentation process. These umami substances can be classified into amino acids, nucleotides, organic acids, and umami peptides, etc., according to their molecular structures and characteristics [3]. In particular, umami peptides (<3000 Da), as a new type of umami substance, have attracted much attention due to their high flavor activity, nutritional value, and the advantages of enhancing umami and reducing salt. They are considered the key contributors to the rich and mellow taste of soybean paste [4,5].

The flavor and quality of soybean paste result from the complex interplay between raw material components and the succession of microbial communities [6]. As one of the most critical raw material components in the fermentation process of soybean paste, soy protein is degraded into small-molecule peptides, amino acids, and other metabolites under the action of hydrolytic enzymes secreted by various microorganisms [7]. These small-molecule products not only serve as the core carriers supplying nitrogen sources for soybean paste but also directly contribute to characteristic flavors such as umami. Therefore, the spatial conformation, aggregation state, and enzymatic digestibility of soy protein directly determine the fermentation efficiency, umami intensity, and overall quality of the soybean paste. However, the dense structure of natural soy protein limits the contact between proteases and their cleavage sites during fermentation, thereby restricting protein hydrolysis efficiency and the generation of umami-conferring compounds. This leads to common issues in traditional soybean paste, such as insufficient umami intensity, unbalanced flavor, and unstable batch quality in traditional soybean paste. Therefore, the targeted structural modification of soy protein to expose more enzyme cleavage sites can significantly improve its enzymatic hydrolysis efficiency and the release of flavor substances during the fermentation process, which is an effective strategy to overcome this bottleneck [8].

Microwave puffing, as a novel physical modification technique, has garnered significant attention due to its advantages of short heating time, operational safety, green efficiency, and ease of industrial application [9,10]. It leverages the rapid penetration and internal heating properties of microwave energy to alter intermolecular forces between proteins, inducing conformational rearrangements that regulate protein aggregation and unfolding behavior [11,12]. This facilitates more efficient degradation of proteins into amino acids and peptides [13]. Bruno et al. [14] demonstrated that microwave and ultrasonic pretreatment enhance the recovery rate, umami component content, and antioxidant activity of LRH protein hydrolysates. Jiang et al. [15] found that brief microwave treatment opens the molecular structure of aggregates in soy protein, thereby enhancing functional properties. Long-term microwave treatment can cause previously depolymerized protein molecules to reform larger molecular aggregates, diminishing functional properties. Sun et al. [16] observed that steam-microwave cooking improved the meat tenderness of braised pork tenderloin and intensified the savory and umami of broth by promoting protein unfolding and degradation. Previous studies have confirmed that microwave treatment can alter protein structure and enhance the release of flavor-related compounds. However, most existing research only focuses on the single effect of microwave on protein structure or the influence of a single quality indicator. To date, there is still a lack of systematic research on how microwave puffing regulates the conformational evolution of soy protein, and the intrinsic relationship between protein structure changes during solid-state fermentation, and the formation of umami peptides remains unclear.

Based on this, this study conducted a gradient-time microwave puffing pretreatment on whole soybeans and comprehensively analyzed the changes in the structure of soy protein. Further, it explored the influence of the optimal microwave puffing conditions on the physicochemical properties and umami potential of koji and fermented soybean paste. The implementation of this study not only provides new technical paths for improving the quality and flavor of soybean paste but also offers theoretical support for the application of microwave puffing technology in the food fermentation industry.

## 2. Materials and Methods

### 2.1. Materials

Northeast soybeans and flour were purchased from Heilongjiang, China. *Aspergillus oryzae* was purchased from Shandong Yishui Jinrun Biotechnology Co., Ltd., China. All other reagents were analytical grade.

### 2.2. Microwave Puffing Treatment of Whole Soybeans

After sorting and dust-removing whole soybeans, they were placed in a microwave oven and treated at a fixed power of 400 W for 30 s, 60 s, 90 s, 120 s, and 150 s, respectively. A total of five treatment groups were obtained, named MSP–30, MSP–60, MSP–90, MSP–120, and MSP–150. Untreated raw soybeans were used as the control and designated as SP. It is worth noting that the 400 W power was selected through preliminary experiments: low power (200–300 W) shows no change in appearance or smell and barely altered protein conformation; power exceeding 400 W (500–600 W) caused soybeans to overheat and burn off-flavors, which is not conducive to subsequent fermentation. The optimal microwave conditions were selected through sensory evaluation and characterization of soy protein structure for the preparation of fermented soybean paste.

### 2.3. Extraction of Soy Protein Isolate

Soy protein isolate was extracted using the alkali extraction and acid precipitation method [17]. The specific procedure is as follows: Natural whole soybeans and whole soybeans subjected to different microwave puffing durations were ground through a 100-mesh sieve, defatted, and dissolved in deionized water at a 1:15 (*w*/*v*) mass ratio of soybean powder to solvent. The pH was adjusted to 8.0 using 2 mol/L NaOH, and the mixture was stirred at low speed in a water bath at room temperature for 1 h. The solution was then centrifuged at 4 °C (8000 r/min, 20 min) to remove the precipitate, and the supernatant was collected. The pH was adjusted to 4.5 using 2 mol/L HCl, and the mixture was allowed to stand for 30 min to precipitate. It was centrifuged again (8000 r/min, 20 min), and the supernatant was discarded. The precipitate was washed three times with distilled water, then dissolved in distilled water. The pH was adjusted to 7.5 to ensure complete dissolution. The freeze-dried soy protein isolate (SPI) fractions were obtained. They were designated SPI, MSPI–30, MSPI–60, MSPI–90, MSPI–120, and MSPI–150 according to their respective microwave treatment times.

### 2.4. Structural Characterization of Soy Protein Isolate

#### 2.4.1. FTIR Spectrum

Following the method of Dong et al. [18], the Fourier transform infrared spectroscopy of BL.Tensor37 (Bruker Corporation, Karlsruhe, Germany) was employed to conduct infrared spectroscopy analysis on the samples. The potassium bromide pellet method was employed: 2 mg of the soy protein sample was thoroughly mixed and ground with 200 mg of KBr powder, then pressed into pellets. Measurements were conducted at (25 ± 0.2) °C with a spectral range of 400–4000 cm^−1^, resolution of 4 cm^−1^, and 32 scans to collect the infrared spectrum.

Spectral Analysis: Curve fitting analysis was performed using PeakFit version 4.12 software to quantify the conformational changes in soybean proteins in the amide I band (1600–1700 cm^−1^). Spectra were subjected to Savitzky–Golay smoothing, linear baseline correction, and Gaussian deconvolution. Second-derivative spectra generated by the software were used to identify the spectral bands of each component, and the analysis results were presented as peak areas at the corresponding wavelengths.

Data Analysis: Origin 8.6 software was used for data plotting and statistical analysis.

#### 2.4.2. UV Absorption Spectrum

UV spectroscopic scanning analysis of soy protein isolate was performed according to the method of Ren et al. [19]. The sample was diluted to 2 mg/mL in PBS buffer (pH 7.0). Using the Lambda 35 UV-Vis spectrophotometer (PerkinElmer, Springfield, IL, USA). The UV spectrophotometer was set to scan wavelengths from 200 to 600 nm at a scan rate of 100 nm/min, with a bandwidth of 5.0 nm, an interval of 1.0 nm, a response time of 0.2 s, an optical path length of 1 cm, and a test temperature of (25 ± 0.2) °C. The scan was repeated three times to obtain the UV absorption spectrum.

#### 2.4.3. Intrinsic Fluorescence Spectrum

Following the method of Xia et al. [20], the fluorescence spectra of the samples were measured using a Hitachi F-7000 fluorescence spectrophotometer (Hitachi, Tokyo, Japan). Samples were dissolved in PBS buffer (0.01 M, pH 7.0) to achieve a final concentration of 0.05 mg/mL. The sample solution was placed in a quartz cuvette. The intrinsic fluorescence spectrum was measured under the following conditions: excitation wavelength 290 nm, emission wavelength range 300–450 nm, slit width 5.0 nm, and scanning speed 10 nm/s.

#### 2.4.4. Surface Hydrophobicity (H_0_)

Surface hydrophobicity of samples was determined using the ANS fluorescence probe method [21]. Protein samples were dissolved in PBS buffer (0.01 M, pH 7.0) at concentrations of 1, 2, 4, 6, and 8 mg/mL. 5 mL of sample solution was taken, to which 50 μL of 8 mmol/L ANS was added. The mixture was vortexed and allowed to stand for 5 min. The excitation wavelength was set to 390 nm (slit correction 5 nm), and the emission wavelength was set to 470 nm (slit correction 5 nm) to measure fluorescence intensity. A curve of fluorescence intensity versus protein concentration was plotted. The slope of the curve during its initial phase represents the protein’s surface hydrophobicity index (H_0_).

#### 2.4.5. Particle Size and Zeta (ζ) Potential

The particle size distribution and Zeta (ζ) potential of the sample were determined using the 90 plus PALS High-Sensitivity Zeta Potential and Particle Size Analyzer (Brookhaven Instruments, Nashua, NH, USA). Using the principle of dynamic light scattering, the sample was diluted to a mass concentration of 1 mg/mL in PBS solution (pH 7.0) and added to the measurement cell. Its particle size distribution characteristics were determined at (25 ± 0.2) °C [22]. The zeta potential of the sample is the average of three measurements. The instrument measures the laser intensity scattered from the sample and then reports the particle size distribution that provides the closest fit between theoretical calculations and experimental measurements.

#### 2.4.6. Scanning Electron Microscopy (SEM)

The morphology of freeze-dried SPI and MSPI samples was observed using a S-3400 scanning electron microscope (Hitachi) at an acceleration voltage of 20 kV. A 15 nm thick platinum coating was deposited onto the sample surface using an ion sputter. Magnification was adjusted, and photographs were captured.

### 2.5. Soybean Paste Preparation

Taking the soybean paste production process in Northeast China as an example, two groups of 250 g whole soybeans each were selected. After sorting and washing, one group underwent microwave puffing (400 W, 90 s) pretreatment before preparing soybean paste, designated as the MSP–90 group. The other group of untreated whole soybeans served as the control group for soybean paste preparation, designated as the SP group. During water soaking, microwave puffing caused the soybean protein to form a porous microstructure, significantly increasing the soybeans’ water absorption rate. Therefore, to ensure consistent moisture content in both groups of soybeans before steaming, the SP soybeans were soaked for 5 h, and the MSP–90 soybeans were soaked for 3.5 h, until the soybeans had fully absorbed water and expanded to twice their original volume, with no wrinkles on the surface and no hard centers inside. The final moisture content (SP: 58.21 ± 0.32%; MSP–90: 57.95 ± 0.28%). There was no significant difference in the hydration status between the two groups before steaming (*p* > 0.05), thereby eliminating the interference of water absorption on subsequent experiments. The water was drained, and steamed at 0.18–0.2 MPa for 5 min. They were then cooled to room temperature, mixed with 60 g of cooked flour, and inoculated with pure *Aspergillus oryzae* culture at 0.2% (*w*/*w*) for koji production. Koji was fermented at 31 °C with ventilation maintained. The prepared soybean koji was transferred to fermentation containers, leveled, lightly compacted, and mixed with 10% brine solution. The mixture was placed in a 42 °C incubator for temperature-controlled fermentation. After 15 days, the fermented mash matured. The temperature was then lowered to 32 °C for an additional 15 days of post-fermentation. The mixture was stirred 1–2 times daily during fermentation.

### 2.6. Physicochemical Properties of Soybean Paste

The spore count of koji samples was measured following the standard protocol SB/T 10315-1999 [23] for spore enumeration. First, 1.0 g of aseptically collected koji sample was placed into a sterile conical flask, supplemented sequentially with 5 mL of 95% (*v*/*v*) ethanol, 20 mL of sterile deionized water, and 10 mL of dilute sulfuric acid. The mixture was vortexed vigorously for 5 min to achieve full dispersion, followed by filtration. The obtained filtrate was quantitatively diluted to 500 mL with sterile deionized water for subsequent spore counting.

Protease activity was determined via the UV spectrophotometric method with casein as substrate, in accordance with GB/T 23527.1-2023 [24]. In brief, 1.0 g of the sample was fully homogenized, transferred to a volumetric flask, and mixed with 50 mL of pH 7.0 buffer solution. The mixture was shaken in an oscillator for 30 min for extraction, and then filtered, and the collected supernatant was adopted as the test solution.

For pH measurement, 5 g of the soybean paste sample was ground in a mortar, diluted with 50 mL of distilled water in a beaker after thorough grinding, and stirred uniformly before filtration. The filtrate pH was tested using a pre-calibrated pH meter.

Total acid and amino acid nitrogen contents were quantified referring to GB 5009.235-2016 [25]. Titration was performed with 0.05 mol/L NaOH standard solution, and the total acid content was calculated according to the NaOH consumed volume. Amino acid nitrogen was determined via the formaldehyde titration method: amino groups of sample amino acids were immobilized by formaldehyde, followed by titration with NaOH standard solution for content calculation.

Small peptide content was determined by the trichloroacetic acid (TCA) method. Large molecular proteins in the sample were precipitated by trichloroacetic acid, and the supernatant was taken after centrifugation. The content of small peptides was determined by the corresponding color reaction.

Reducing sugar content was determined by the 3,5-dinitrosalicylic acid (DNS) colorimetric method. Reducing sugars reacted with the DNS reagent in a boiling water bath to generate brownish-red products. The absorbance of the reaction solution was measured via spectrophotometry, and the reducing sugar content was calculated based on the established standard curve.

Sample moisture content was tested through the direct drying method specified in GB 5009.3-2016 [26], with constant-temperature drying conducted in a 105 °C oven.

The color parameters (L*, a*, and b*) of the soybean paste were measured using an LS173 colorimeter (Shenzhen Linshang Technology Co., Ltd., Shenzhen, China). All determinations were performed in triplicate, and average values were taken as the final results.

### 2.7. Determination of Peptide Sequences in the Water Extract of Soybean Paste

The peptide components in the water extracts of MSP–90 and SP soybean paste after 30 days of fermentation were analyzed by LC-MS/MS. A Thermo Fisher Scientific EASY-nLC 1200 liquid chromatograph equipped with a C18 column was used (Thermo Fisher, Waltham, MA, USA). The specific steps were as follows: the samples were added with water, thoroughly shaken to dissolve, and then subjected to reduction, alkylation, and desalination before being analyzed on the machine. All detected peptides are natural fermentation products. During the pretreatment process, no peptide bonds will be cleaved, nor will new peptide fragments be generated. The sample was injected, with a flow rate controlled at 600 nL/min, an electrospray voltage of 2 kV, and a column temperature of 55 °C. The mobile phase A was a 0.1% formic acid aqueous solution, and the mobile phase B was a 0.1% formic acid solution in 80% acetonitrile. The chromatographic gradient was set as follows: 0–66 min, B phase 4–95%. The mass spectrometer was operated in data-dependent acquisition mode and automatically switched between MS and MS/MS modes. The parameters were set as follows: (1) MS: scan range (*m*/*z*) = 100–1500; resolution = 70,000; automatic gain control maximum injection time = 100 ms; (AGC) target = 3 × 10^6^; (2) HCD -MS/MS: resolution = 17,500; scan range (m/z) = 100–2000; maximum injection time = 100 ms; (AGC) target = 1 × 10^5^. The raw mass spectrometry results were obtained and compared with the UniProtKB database (release 2026_02) for analysis to obtain the peptide sequence structure in the soybean paste.

### 2.8. Evaluation of the Taste Characteristics of Soybean Paste

#### 2.8.1. Sensory Evaluation

All sensory evaluation studies were approved by the Science and Technology Ethics Committee of Qiqihar University (Approval number: 20250921). The sensory panel consisted of 20 volunteers (10 male, 10 female, aged 20–30), all recruited from the College of Food and Biotechnology at Qiqihar University (Qiqihar, China). It should be noted that the sensory results are only applicable to the 20–30-year-old population in this study and do not represent the taste preferences of all consumer groups. All participants provided informed consent to take part in this sensory study. Prior to the formal experiment, all panel members received standardized training on taste identification, scoring criteria, and sample evaluation, meeting the sensory assessment standards: no history of allergies or taste disorders, and substantial prior experience in sensory evaluation. This study employed a ten-point scale (0 = none, 10 = extremely strong) to conduct quantitative descriptive sensory analysis of untreated whole soybeans and whole soybeans subjected to microwave puffing under 400 W power for varying durations (30, 60, 90, 120, and 150 s). Evaluation attributes included color, texture, aroma, scorched flavor, and surface integrity. All evaluations were conducted in a sensory laboratory compliant with international standards. Samples were randomly assigned three-digit anonymous codes and presented in a double-blind, randomized order to eliminate subjective bias. To minimize sensory fatigue during testing, participants were instructed to rinse their mouths thoroughly with water and rest for five minutes after evaluating each sample.

The sensory evaluation method for the two soybean paste samples (MSP–90 and SP) with a 30-day fermentation period was conducted as described above. In accordance with the requirements of the Chinese national standard GB/T 24399–2009 [27], umami, sweetness, saltiness, sourness, and bitterness were selected as the core evaluation indicators. The soybean paste samples were taken out of the refrigerator and allowed to equilibrate at room temperature (25 ± 0.2 °C) before sensory evaluation. All sensory data were ultimately analyzed statistically and visualized using Origin software.

#### 2.8.2. Electronic Tongue Determination of Soybean Paste

A comprehensive taste analysis was performed using an E-tongue instrument (SA402B, Insent, Tokyo, Japan). Prior to testing, the E-tongue was calibrated using standard solutions in accordance with the manufacturer’s instructions. First, the positive and negative electrodes were immersed in the positive and negative cleaning solutions, respectively, for 90 s. They were then rinsed with a reference solution for 120 s, followed by another reference solution for 120 s. Testing began 30 s after the sensor returned to its equilibrium position and lasted 30 s. The sensor was then rinsed for 3 s in each of the two sets of reference solutions, after which it was immersed in a new reference solution to test for aftertaste for 30 s. Drift correction and sensor self-cleaning were performed between each sample test to ensure data reliability. Weigh 24.00 g of the soybean paste sample, grind it into a paste, and mix it with 120 mL of distilled water. Stir thoroughly in a 50 °C water bath for 1 h, then filter. The supernatant was collected and loaded into the E-tongue autosampler. Specific taste sensors (umami, salty, sweet, sour, and bitter) were selected. After processing by the pattern recognition system, the collected taste information was transmitted to the data acquisition processor. Following software analysis, the corresponding taste data was obtained. To minimize system error, raw data from three measurements were used for analysis.

### 2.9. Statistical Analysis

All experiments in this study adopted three independent biological replicates (3 separate soybean paste fermentation batches). For each fermentation batch, all index determinations were performed with 3 technical replicates (instrument repeated measurements). The final data were presented as mean ± standard deviation of three biological replicates. Data were statistically analyzed using Duncan’s multiple range test (*p* < 0.05), and Origin software was employed for graphing, data processing, and statistical analysis.

## 3. Results and Discussion

### 3.1. Selection Conditions for Optimal Microwave Puffing of Whole Soybeans

**Figure 1:**
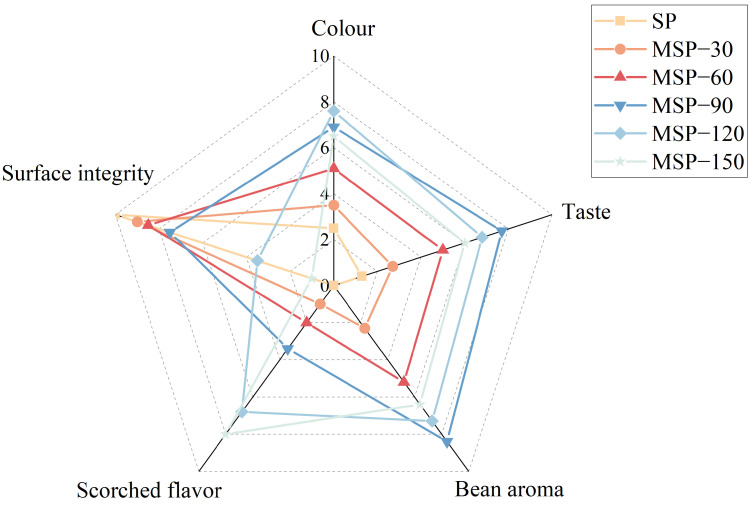
Radar chart of sensory analysis of whole soybeans under different microwave puffing times (0 s, 30 s, 60 s, 90 s, 120 s, and 150 s).

Quantitative sensory evaluations of SP (untreated) and MSP samples subjected to different microwave puffing times (30, 60, 90, 120, 150 s) were conducted across five dimensions: color, taste, bean aroma, scorched flavor, and surface integrity. Results are presented in Figure 1. The figure indicates that when microwave puffing treatment time ≤90 s, the scores for bean aroma, crispness, and color increased with longer processing times, with color gradually deepening from light yellow to golden yellow. At 90 s, the overall sensory score peaked, characterized by a rich bean aroma, crisp texture, golden yellow color, minimal off-flavors, and good surface integrity. When processing time exceeded 90 s, further microwave exposure caused significant declines in bean aroma and taste scores. Undesirable flavors like scorched increased markedly, color shifted to burnt yellow, and bean cracking intensified. Surface integrity scores decreased, leading to overall sensory quality deterioration. In summary, microwave puffing at 400 W for 90 s represents the optimal treatment condition for whole soybeans. Under this condition, the sample exhibits the best performance in color, taste, bean aroma, scorched flavor, and surface integrity. To clarify the impact of this microwave puffing condition on the quality of the subsequent fermented soybean paste, further analysis of soybean protein structural changes is required.

### 3.2. Effects of Microwave Puffing on Soy Protein Conformation and Microstructure

#### 3.2.1. FTIR Spectroscopy Analysis

**Figure 2:**
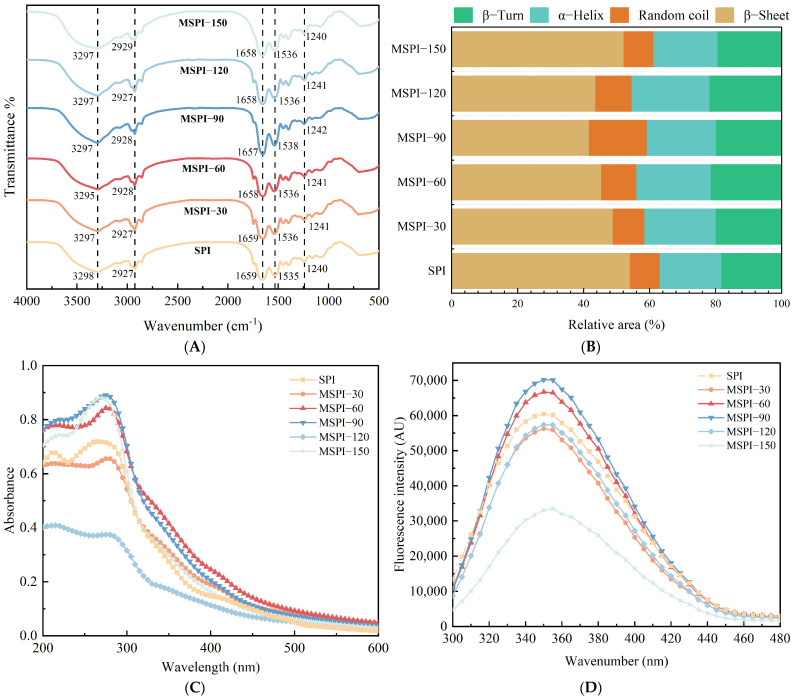
Spectral diagrams of SPI and MSPI under different microwave puffing treatment times (30 s, 60 s, 90 s, 120 s, and 150 s): (**A**) FTIR spectrum; (**B**) the secondary structure content; (**C**) UV spectrum; and (**D**) intrinsic fluorescence spectra.

Fourier transform infrared spectroscopy is commonly employed to detect changes in protein secondary structure [28]. Infrared spectroscopy was used to analyze the secondary structure of SPI and MSPI, as shown in Figure 2A. It can be observed that no new characteristic peaks appeared in the soy proteins after microwave puffing treatment. SPI exhibits a broad characteristic absorption peak at 3297 cm^−1^, attributed to the stretching vibrations of O–H and N–H bonds. Compared to SPI, MSPI shows a slight red shift, reflecting weakened C=O···H-N hydrogen bonds within the soy protein molecules and intermolecular H–O···N-H hydrogen bond interactions. A short, sharp absorption peak at 2927 cm^−1^ is attributed to the C–H stretching vibrations of –CH_3_ and –CH_2_ groups in the saturated structures of soy protein molecules, reflecting the asymmetric C–H stretching vibrations in the aliphatic amino acid side chains. The 1800–1000 cm^−1^ region constitutes the fingerprint zone of soy protein infrared spectra [29]. Compared to SPI, MSPI, particularly MSPI–90, exhibits red shifts in absorption peaks at 1659 cm^−1^, attributed to the C=O stretching vibration of amides within the amide I band (1700–1600 cm^−1^). This phenomenon indicates the disruption of hydrogen bonds stabilizing the α–helical structure, leading to the breakdown of the hydrogen bond network and increased freedom of movement in the peptide chain. The absorption peak at 1535 cm^−1^ in MSPI–90 exhibited a blue shift, attributed to the N-H bending and C-N stretching vibrations within the amide II band (1600–1500 cm^−1^). This indicates that during denaturation, the β–turn structure undergoes conformational compression, with the N–H bond angle being spatially squeezed by adjacent residues and partially converting to an extended conformation. The absorption peak at 1240 cm^−1^ corresponds to amide III (1330–1220 cm^−1^), primarily involving C–N stretching and N–H out-of-plane bending vibrations. Collectively, these findings indicate significant alterations in the secondary structure of soy protein molecules following microwave puffing treatment.

According to previous reports, the amide I band (1700–1600 cm^−1^) in infrared spectroscopy is a sensitive region for soy protein secondary structure, comprising various secondary structures such as α–helices, β–sheets, β–turns, and random coil. It is the primary spectral band for calculating protein secondary structure [30,31]. To extract additional information, the amide I band (1700–1600 cm^−1^) underwent deconvolution and curve fitting. The correspondence between each subpeak and secondary structure is as follows: the absorption peak region for α–helix spans 1648–1664 cm^−1^, β–sheet absorption peaks span 1615–1637 cm^−1^ and 1682–1700 cm^−1^, β–turn absorption peaks range from 1664 to 1681 cm^−1^, and random coil absorption peaks extend between 1637 and 1648 cm^−1^ [32]. Figure 2B shows the quantitative analysis of the secondary structure of SPI and MSPI. In SPI, β–sheet structures accounted for the highest proportion at 54.13%, while random coil had the lowest proportion at 9.03%. Interestingly, the secondary structure composition of soy protein underwent significant changes after microwave puffing. Compared to SPI, the most pronounced changes in MSPI–90’s secondary structure were a decrease in β–sheet content and an increase in random coil. The relative β–sheet content decreased by 12.41%, while the relative random coil content increased by 8.46%. Meanwhile, the α–helix and β–turn contents showed slight increases, rising by 2.32% and 1.62%, respectively. These results indicate that the β–sheet structure was largely converted into random coil and a small amount of α–helix and β–turn structures. This phenomenon occurs because β–sheet structures are typically buried within the polypeptide chain. Microwave puffing treatment induces high-frequency oscillations between dipole molecules and polar side chains within soy protein isolate molecules, breaking some hydrogen bonds. This locally extends amino acid sequences, altering the protein’s spatial conformation and loosening its structure. The increased content of random coil further confirms the formation of more disordered molecular structures during microwave puffing. The slight increase in α–helix and β–turn structures may result from the exposure of hydrophobic residues buried within the molecule during microwave puffing treatment. This exposure allows partial unfolding and intermolecular cross-linking, forming a small amount of α–helix and β–turn structures.

In summary, microwave puffing treatment partially disrupts the inherent rigid structure of SPI, increasing its flexibility. This transforms the protein conformation from ordered to disordered and exposes hydrophobic groups. These trends were previously reported in prior studies [33,34]. Although we have only observed quantitative changes in secondary structure, it can be inferred that tertiary structure also undergoes certain modifications. For instance, tightly coiled tertiary structures may become more extended, allowing deeply buried hydrophobic groups within the molecule to be exposed.

#### 3.2.2. UV Absorption Spectra Analysis

Ultraviolet absorption spectroscopy is a common analytical method for studying protein conformational changes. Changes in the wavelength and absorption intensity of characteristic peaks of functional groups can be used to evaluate structural alterations in proteins [35]. Figure 2C showed the UV absorption spectra of SPI and MSPI. The figure reveals that SPI exhibits a strong absorption peak at 260–280 nm. This peak primarily originates from the π→π* transition of aromatic amino acid residues such as tryptophan (Trp) and tyrosine (Tyr), forming an absorption band due to the chromophore’s absorption of UV. Following different microwave puffing treatment times, the UV absorption intensity of MSPI exhibited changes. Among them, the UV absorption peak intensity of MSPI–90 is the strongest. This may result from microwave puffing altering the spatial conformation of soy protein molecules. The increased polarity in the microenvironment surrounding aromatic ring hydrophobic groups and the greater extension of peptide chain structures modify the exposure levels of surface chromophores (Tyr and Trp), thereby shifting UV absorption peaks or altering peak shapes. MSPI–90 exhibits the greatest exposure of Tyr and Trp, suggesting the most pronounced unfolding of the protein’s tertiary structure. However, prolonged microwave puffing treatment may reduce protein solubility, increase aggregation of chromophores and hydrophobic groups, and decrease exposure of chromophore amino acids, leading to reduced UV absorption intensity.

#### 3.2.3. Intrinsic Fluorescence Spectroscopy Analysis

Endogenous fluorescence spectroscopy is commonly employed to detect conformational changes in protein tertiary structures. It primarily reflects the fluorescence emission of aromatic amino acids, which are highly sensitive to alterations in the protein’s microenvironment and are closely associated with its folding state [36]. When proteins are excited by light at 290 nm, the resulting fluorescence emission is primarily attributable to the characteristic fluorescent responses of Trp and Tyr residues. Consequently, the unfolding of protein molecules following microwave puffing can be quantified by measuring the fluorescence intensity at these residual sites in protein samples, thereby providing a more accurate prediction of changes in protein tertiary structure [37,38].

Figure 2D shows the fluorescence spectra of SPI and MSPI. It can be observed that both SPI and MSPI exhibit a maximum absorption wavelength (λmax) at 354 nm. However, microwave puffing treatment caused significant changes in the fluorescence intensity of soy protein isolate at this wavelength. Compared with the SPI, the endogenous fluorescence intensity of MSPI generally showed an initial increase followed by a decrease, with MSPI–90 exhibiting the highest fluorescence intensity. This trend aligns with the results from ultraviolet spectroscopy testing. This may result from microwave puffing breaking non-covalent bonds within soy protein, such as hydrophobic interactions or hydrogen bonds. This leads to a more extended protein structure, exposing internal Trp and Tyr residues to a polar environment [39]. However, as microwave puffing treatment time increased, the fluorescence intensity decreased and was slightly lower than that of SPI. Based on prior research [40], this might be due to the long-term microwave puffing treatment causing the soy protein to reaggregate through intermolecular interactions and secondary structure transformations. Consequently, exposed tryptophan residues on the protein surface become re-encapsulated, diminishing fluorescence intensity. These results indicate that the internal structure of MSPI exhibits maximum extension at 90 s of microwave puffing.

#### 3.2.4. Surface Hydrophobicity (H_0_) Analysis

**Figure 3:**
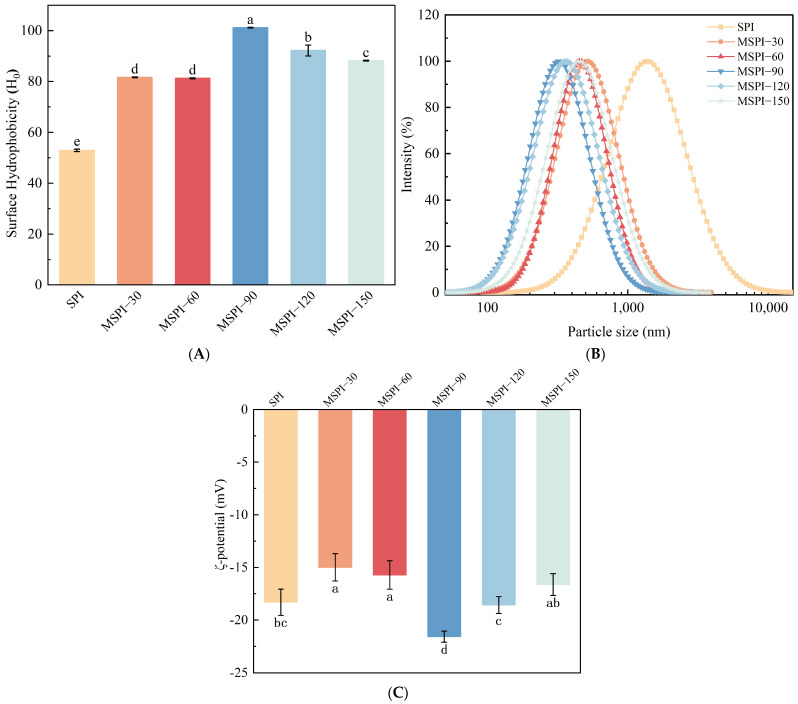
(**A**) Surface hydrophobicity, (**B**) particle size distribution, and (**C**) ζ–potential of SPI and MSPI under different microwave puffing treatment times (30 s, 60 s, 90 s, 120 s, and 150 s). Different letters in the graphs indicate that the results are significantly different (*p* < 0.05).

Protein surface hydrophobicity (H_0_) is a key parameter widely used to evaluate changes in protein structure and function, as well as emulsifying properties [41]. Figure 3A shows the surface hydrophobicity results of SPI and MSPI. It indicates that the H_0_ of MSPI significantly increased after microwave puffing treatment (*p* < 0.05), compared with that of the SPI. As microwave puffing treatment time increased, the H_0_ of MSPI generally followed a pattern of initial increase followed by a decrease, peaking at 90 s. This indicates that during the microwave puffing treatment of SPI, the initially dominant non-thermal effects induced collisions between MSPI molecules and molecular vibrations, leading to the breaking of non-covalent bonds represented by hydrophobic interactions. This resulted in the disruption of protein aggregate structures and the exposure of hydrophobic regions on the protein surface. Additionally, appropriately low-intensity thermal effects also promote the unfolding of protein structures, exposing hydrophobic groups originally buried internally and thereby increasing the protein’s surface hydrophobicity. However, as microwave puffing treatment time continues to increase, thermal effects become increasingly strong and dominant. In MSPI, polar groups such as carbonyl groups gradually increase and undergo cross-linking through hydrophobic interactions, re-embedding hydrophobic groups within the protein interior, resulting in reduced surface hydrophobicity.

#### 3.2.5. Size Distribution and Zeta (ζ) Potential Analysis

The particle size distribution of proteins provides a macroscopic reflection of aggregation and disaggregation phenomena occurring within protein molecules under physical field influences [42]. Figure 3B shows the average particle size distribution results for SPI and MSPI. Compared with the SPI, the particle size of MSPI significantly decreased after microwave puffing. As microwave puffing treatment time increased, the MSPI particle size gradually shifted toward smaller particles, with the most pronounced reduction occurring at 90 s. After 90 s, as microwave puffing treatment time continued to increase, the MSPI particle size distribution gradually shifted to the right, indicating an increase in particle size. This phenomenon likely occurs because short-term microwave puffing treatment promotes molecular motion in soy protein isolate through non-thermal effects such as electric fields, increasing the probability of intermolecular collisions. This leads to the breaking of non-covalent bonds between soy protein isolate molecules, causing the disaggregation of larger aggregate molecules into smaller aggregates. However, as microwave puffing treatment time further increases, collisions among soy protein isolate aggregates intensify. Concurrently, enhanced thermal effects within the system induce the denaturation of already depolymerized aggregate molecules. This process forms new chemical bonds, strengthens intermolecular interactions, and ultimately leads to the formation of larger aggregates, resulting in increased MSPI particle size. This trend aligns with findings reported by Wang et al. in their study on the effects of microwaves on the aggregate structure of oxidized soy protein [43].

Combined surface hydrophobicity results reveal a highly significant linear negative correlation between soy protein isolate surface hydrophobicity and particle size. This indicates that soy protein isolate surface hydrophobicity decreases as particle size increases. The broad particle size distribution of the protein indicates that protein molecules aggregate to form numerous clusters. This embedding of hydrophobic groups reduces the exposure of surface hydrophobic groups, leading to decreased surface hydrophobicity. Conversely, when fewer aggregates form between protein molecules, the particle size distribution narrows, allowing more hydrophobic groups to be exposed on the protein surface, thereby increasing surface hydrophobicity.

The zeta-potential correlates with the charge density on the protein surface [44]. Soy protein isolate is a typical ampholytic electrolyte containing numerous polar groups (e.g., carboxyl, hydroxyl, and amino groups) and nonpolar groups on its side chains. These hydrophilic polar groups are exposed on the soy protein isolate surface in aqueous solution, imparting surface charge to the protein. Measuring the zeta potential of soy protein isolate solutions is highly informative for characterizing their solution properties [45]. Figure 3C shows the zeta potential profiles of SPI and MSPI. As evident from the Figure 3C, both SPI and MSPI exhibit negative zeta potentials, indicating a preponderance of negative charges on the protein surface. Specifically, the number of negatively charged amino acids outweighs positively charged ones, resulting in negative zeta potentials for protein solutions. As microwave puffing treatment time increases, the absolute value of MSPI’s zeta potential first rises and then decreases, peaking at 90 s of microwave puffing treatment. This changing trend is similar to surface hydrophobicity. This may occur because during short-term microwave puffing, the electromagnetic field alters the electrostatic equilibrium of the protein solution. This increases the number of like charges on the MSPI surface. The enhanced electrostatic repulsion between like charges weakens intermolecular aggregation, reducing the internal folding of hydrophobic groups. This exposes more hydrophobic groups on the molecular surface, increasing the protein’s surface hydrophobicity. As microwave puffing treatment time increased to 90 s, the absolute value of MSPI’s zeta potential gradually decreased. This may result from polar dipolar molecules continuously oscillating within the microwave electromagnetic field, generating substantial heat that directly acts upon MSPI. This process reduces the number of like charges on its surface. The diminished electrostatic repulsion decreases solution stability. Protein molecules in the system tend to aggregate. This causes the hydrophobic groups of proteins to become buried within the aggregates due to molecular clustering, reducing the hydrophobicity of the protein surface. This trend is consistent with previous studies [46].

#### 3.2.6. SEM Analysis

**Figure 4:**
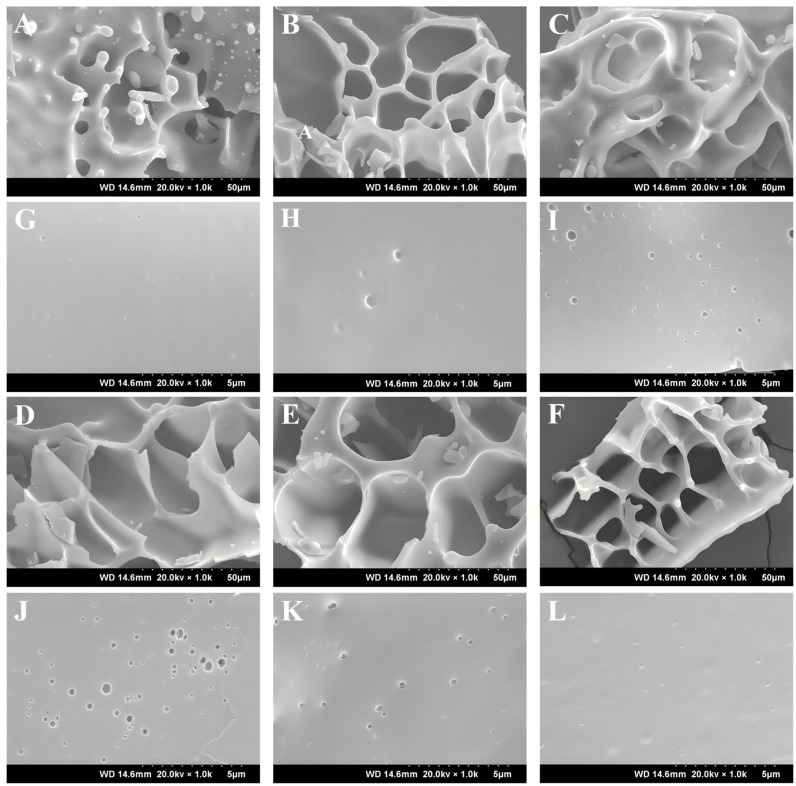
Microstructure of lyophilized SPI (**A**,**G**) and MSPI (**B**–**L**) determined by scanning electron microscopy. (**A**–**F**) represent the images of the samples expanded 1000×, corresponding to microwave times of 0 s, 30 s, 60 s, 90 s, 120 s, and 150 s, respectively. (**G**–**L**) represent the images of the samples expanded 10,000×, corresponding to microwave times of 0 s, 30 s, 60 s, 90 s, 120 s, and 150 s, respectively.

Scanning electron microscopy (SEM) analysis is a method for examining the microscopic morphology of objects and observing structural features, directly reflecting the structure and morphology of substances [47]. To further investigate the effect of microwave puffing treatment on protein structure, SEM observations were conducted on SPI and MSPI samples magnified at 1000× and 10,000×, respectively, with results shown in Figure 4. Figure 4A,G present SEM images of SPI magnified at 1000× and 10,000×, respectively. The SPI structure appears relatively compact with a flaky surface and few pores. Figure 4B–L illustrates the structural diagrams of MSPI magnified at 1000× and 10,000× after different microwave puffing times. Compared to SPI, MSPI exhibits distinct surface morphologies following microwave puffing. In Figure 4B,C, as microwave puffing treatment time increases, the MSPI protein matrix gradually develops a porous network structure that becomes increasingly loose and embedded with small fragments. In Figure 4H, I, the originally smooth surface begins to develop pores that progressively multiply. Notably, at 90 s of microwave puffing, these network structures are disrupted, and protein chains unfold (Figure 4D). Figure 4J shows the matrix surface exhibiting numerous pores accompanied by cracks. However, with further microwave puffing, the fragmented network structures recrosslinked to form new aggregates with a more uniformly distributed network structure (Figure 4E,F). In Figure 4K,L, the pores on the matrix surface gradually diminished. This phenomenon aligns with particle size analysis results. It indicates that appropriate microwave puffing treatment can break down macromolecular aggregates into smaller ones through oscillation and other effects. Prolonged microwave puffing induces thermal effects, leading to cross-linking between soy protein isolates via disulfide bonds and hydrophobic interactions, forming larger, more regular aggregates. This finding is consistent with the research by Teng et al. [48].

Based on the comprehensive analysis of sensory evaluation and structural characterization of soybean isolate (MSPI) after different microwave puffing pretreatments, the optimal condition was determined to be 400 W for 90 s. Soybeans treated under this microwave condition and untreated soybeans were used to prepare soybean paste according to the method described in Section 2.5, and their effects on the physicochemical properties and umami of the fermented soybean paste were examined. Notably, during soybean soaking, the microwave puffing pretreatment group was able to reduce the soaking time from 5 h to 3.5 h while maintaining no significant difference in hydration levels before cooking between the two groups—a prominent industrial advantage of this physical modification technique. Microwave puffing creates a porous microstructure in soybean protein, accelerating water penetration into the soybean tissue. This not only ensures uniform substrate conditions for steaming and fermentation but also significantly shortens the raw material pretreatment time, thereby improving the overall production efficiency of soybean paste. These advantages suggest that microwave puffing technology has promising prospects for application in the industrial-scale production of soybean paste and other fermented soybean products.

### 3.3. Effects of Microwave Puffing Pretreatment on the Physicochemical Properties of Fermented Soybean Paste

#### 3.3.1. Effect of Microwave Puffing Pretreatment on Koji-Making

**Figure 5:**
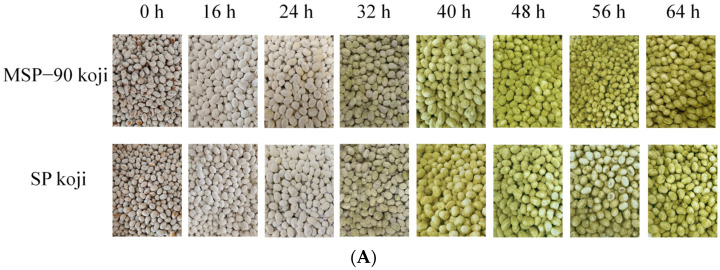

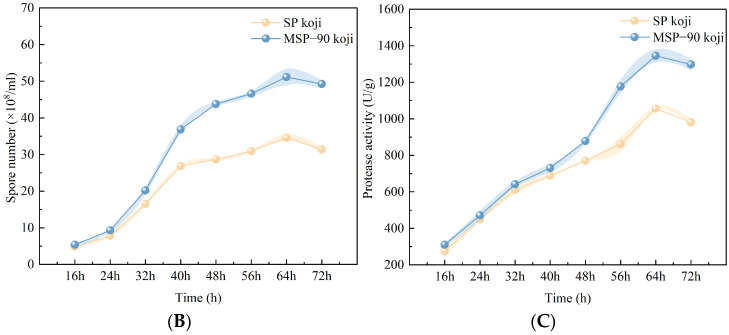
Influence of microwave puffing pretreatment on the growth of koji: (**A**) the appearance; (**B**) spore numbers; and (**C**) proteinase activity. Error bars represent standard errors of three measurements.

*Aspergillus oryzae* was inoculated at 0.2% (*w*/*w*) onto the surfaces of microwave puffing (400 W, 90 s) pretreatment and untreated soybeans mixed with cooked flour. After inoculation, the samples were placed in a 31 °C incubator for constant-temperature koji fermentation. The growth of *Aspergillus oryzae* mycelium and spore production on the koji surface were monitored throughout the fermentation process. As shown in Figure 5A, *Aspergillus oryzae* exhibited slow growth from 0 to 24 h. This phase progressed through three stages: spore germination, initial mycelial growth with slight whitening of the koji surface, and mycelial proliferation with near-complete whitening of the surface. By 32 h, pale yellow to light yellow spores were clearly observable, marking the spore attachment phase. At 40 h, spore production became highly evident with abundant yellow-green spores. The spore production rate in MSP–90 koji samples was significantly faster than in the SP koji samples. Cultivation ceased at 64 h, concluding the koji production process.

In the production of soybean paste, the performance of the microbial strain significantly influences the color, aroma, taste, texture, and raw material utilization rate of the paste, making it crucial for the quality of the finished product [49]. The spore count in starter cultures directly reflects their maturity and serves as a vital indicator of starter quality. As shown in Figure 5B, both seed koji exhibited relatively low spore counts during the initial 24 h of fermentation. During this phase, conidia in the *Aspergillus oryzae* powder germinate to form hyphae. These hyphae continue growing and intertwine to form a mycelium network, where *Aspergillus oryzae* hyphae begin secreting various metabolic enzymes. After 24 h, spore counts in both seed koji increase rapidly. The seed koji turns yellowish-green, and protease activity significantly increases. At 32 h into koji making, the spore count in MSP–90 koji reaches the 10^9^ order of magnitude, significantly exceeding that of SP koji. Spore counts peak at 64 h, with values of 5.11 ± 0.23 × 10^9^/g and 3.46 ± 0.09 × 10^9^/g for the two koji types, respectively. Subsequently, due to nutrient depletion and metabolic product accumulation, the koji entered a decline phase. Koji harvested at 64 h met the national quality standards for soybean paste production. Research indicates that premature harvesting may result in incomplete enzyme system activation, while delayed harvesting can lead to autolysis of spores, releasing intracellular protease inhibitors, which may hinder subsequent fermentation [50]. Therefore, it is most appropriate to harvest the koji at the 64 h fermentation stage.

The number of spores in the seed koji is closely related to the level of protease activity. The vigor of spore germination and mycelial growth directly influences protease secretion, and protease is the core enzyme preparation that degrades soybean protein and generates umami compounds during soybean paste fermentation. Its activity is a key indicator in soybean paste production. Figure 5C shows the protease activity of MSP–90 koji and SP koji over fermentation time. It can be seen from the Figure 5C that the protease activity in the two types of seed koji generally shows a trend of first rising and then falling, which is consistent with the growth trend of spore numbers. At 64 h, protease activity in both types of koji reached its peak, with MSP–90 koji consistently exhibiting higher activity than SP koji. This difference likely stems from microwave-puffed soybeans, where microwave thermal effects rupture cell walls and disrupt cellulose structures, providing accessible carbon sources for mycelial growth. Furthermore, microwave puffing treatment denatures soybean proteins. Thermal processing disrupts secondary and tertiary protein structures, breaking hydrogen bonds, disulfide bonds, and hydrophobic interactions. This transforms dense structures into loose, porous ones, exposing more protease active sites. This not only provides abundant nitrogen sources for mycelial growth but also increases the enzyme–substrate contact area. Studies have shown that “a porous structure is more conducive to *Aspergillus oryzae* mycelium growth, mycelium network formation, and spore germination” [51]. The mycelium of *Aspergillus oryzae* forms a dense network through its porous structure, providing physical support and nutrient pathways for the mycelium. This structure facilitates rapid mycelium expansion and uniform distribution throughout the fermentation substrate, promoting spore germination and mycelium growth. Simultaneously, the loosening of protein structures exposes more enzymatic cleavage sites, significantly enhancing protease degradation and improving enzymatic hydrolysis efficiency. This observation is corroborated by subsequent peptide content results. After 64 h of making the koji, protease activity in the seed koji exhibited a declining trend. Therefore, based on protease activity as an indicator, 64 h was determined to be the optimal time for harvesting the koji. Following koji making, a 10% saline solution was added, and fermentation proceeded for 30 days. The soybean paste fermentation is then complete.

#### 3.3.2. Effects of Microwave Pretreatment on the Physicochemical Properties of Fermented Soybean Paste

**Figure 6:**
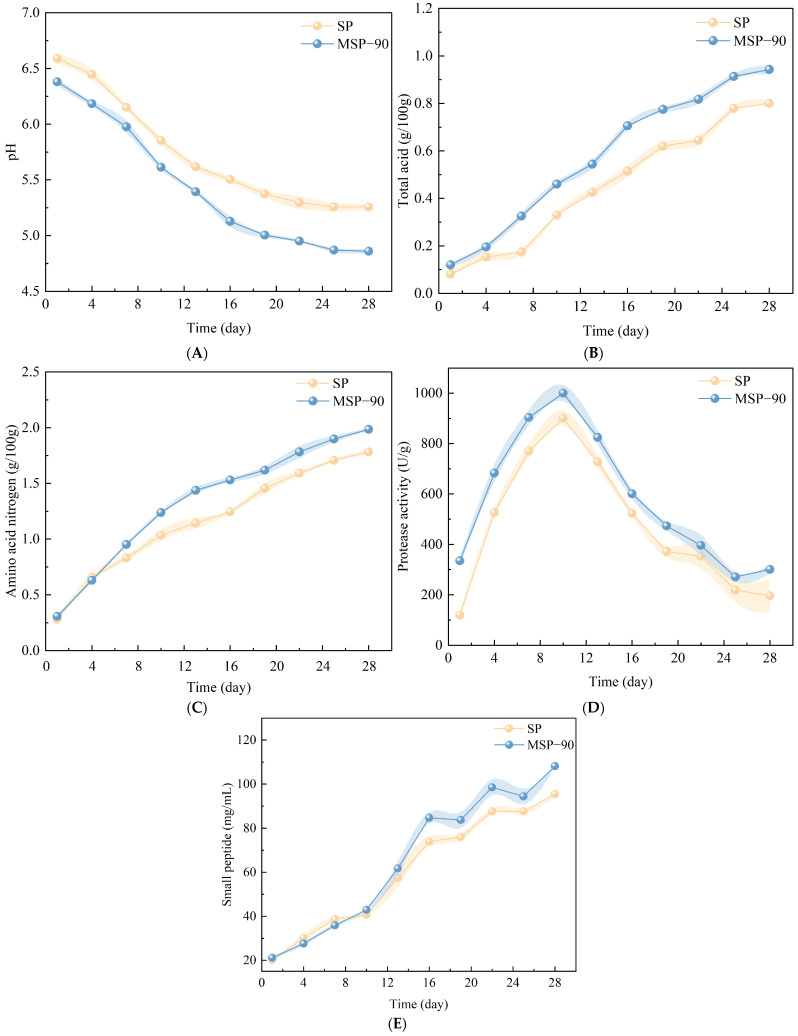
Physicochemical properties of MSP–90 and SP fermented soybean paste: (**A**) pH value; (**B**) total acidity; (**C**) amino nitrogen content; (**D**) protease activity; and (**E**) small peptides content. Error bars represent standard errors of three measurements.

pH, total acid, amino acid nitrogen, protease activity, and small peptide content are key indicators for characterizing the fermentation process of soybean paste, evaluating its quality and umami characteristics [52]. This study systematically analyzed the quality characteristics of soybean paste during fermentation in the microwave puffing pretreatment group (MSP–90) and the untreated control group (SP). The results are shown in Figure 6. Overall, microwave puffing (400 W, 90 s) significantly improved the physicochemical indicators of soybean paste during fermentation. As shown in Figure 6A, the pH of both the MSP–90 and SP groups of soybean paste decreased continuously with the extension of fermentation time, and the pH of the MSP–90 group was consistently lower than that of the SP group throughout the fermentation cycle. At the end of fermentation, the pH of the MSP–90 group dropped to 4.87 ± 0.02, significantly lower than 5.26 ± 0.03 of the SP group (*p* < 0.05). Maintaining the pH of the fermentation system within the range of 4–5 can effectively inhibit the growth and reproduction of pathogenic bacteria, ensuring the safety of the fermented product [53]. Correspondingly, the total acid content of both groups increased continuously with fermentation time (Figure 6B), and the total acid accumulation rate of the MSP–90 group was significantly higher than that of the SP group. At the end of fermentation, the total acid content of the MSP–90 group reached 0.94 ± 0.02 g/100 g, an increase of 17.50% compared to 0.80 ± 0.01 g/100 g of the SP group, indicating that microwave puffing (400 W, 90 s) pretreatment significantly promoted the generation of organic acids during fermentation and accelerated the acidification process of the system.

The content of amino acid nitrogen is a core indicator for characterizing the protein degradation and umami potential of soybean paste [54]. As shown in Figure 6C, the content of amino acid nitrogen in both groups of samples steadily increased with the fermentation time. The increase in the MSP–90 group was significantly higher than that in the SP group, reaching 1.99 ± 0.02 g/100 g at the end of fermentation, which was 11.80% higher than that in the SP group. Moreover, the content of amino acid nitrogen in both groups of samples was much higher than the national standard minimum limit of 0.5 g/100 g stipulated in GB/T 24399-2009 “Soybean Paste”. Previous studies have confirmed that the higher the content of amino acid nitrogen, the higher the amino acid composition in soybean paste, and the more prominent the umami quality [55]. The dynamic changes in protease activity during fermentation (Figure 6D) further explain this phenomenon. The protease activity in both groups of samples showed a trend of first increasing and then decreasing. The MSP–90 group reached its peak (1000 U/g) on the 10th day of fermentation, which was significantly higher than that of the SP group (900 U/g) at the same time, and the protease activity in the MSP–90 group remained at a higher level throughout the fermentation period. The reason for this is that microwave puffing pretreatment makes the originally ordered and compact structure of soy protein become loose and stretched, exposing more enzyme action sites, providing favorable conditions for the efficient enzymatic hydrolysis of protease. The change pattern of small peptide content was consistent with that of amino acid nitrogen (Figure 6E). The rate of small peptide generation in the MSP–90 group was significantly higher than that in the SP group, reaching 108.15 ± 0.47 mg/mL at the end of fermentation, which was 13.30% higher than that in the SP group. This indicates that microwave pretreatment can simultaneously promote the degradation of large molecular proteins in soybeans into small molecular flavor precursors such as peptides and free amino acids.

The physicochemical indicators of the finished products of the two groups of soybean paste (MSP–90 and SP) after fermentation are shown in Table 1. The reducing sugar content of the MSP–90 group was 3.69 ± 0.19 g/100 g, which was significantly increased by 14.95% compared to the SP group. This was attributed to the thermal effect of microwave instantaneous high temperature, which disrupted the ordered structure of starch and promoted starch gelatinization, enhancing the hydrolysis efficiency of the saccharifying enzyme on carbohydrates, and facilitating the generation of reducing sugars. Reducing sugars are not only important precursors for the synthesis of flavor substances in soybean paste, but also provide sufficient carbonyl donors for the Maillard reaction. Moreover, the moisture content of the MSP–90 group was significantly lower than that of the SP group. The lower moisture environment can effectively inhibit the growth of miscellaneous bacteria and reduce the risk of spoilage and deterioration during the storage of fermented soybean paste [56]. Color is an important evaluation index for the sensory quality of soybean paste, and it can also indirectly reflect the progress and intensity of the Maillard reaction in the system. The color results of the soybean paste in Table 1 show that the brightness value L* and redness value a* of the MSP–90 group are significantly higher than those of SP. Among them, the L* value reflects the brightness of the sample’s appearance, and the higher the value, the better the glossiness of the sample. The a* value reflects the depth of the sample’s red tone, and the higher the positive value and the higher the value, the deeper the redness. To a certain extent, it also indicates that the fermentation and metabolic processes are more complete [57]. It can be seen that microwave puffing pretreatment makes the color of the soybean paste redder and brighter. This is mainly attributed to the microwave puffing pretreatment destroying the intact structure of the soybean cell wall, promoting the release and dissolution of pigment precursors such as flavonoids and phenols, and providing sufficient amines and carbonyl donors for the Maillard reaction, promoting the reaction to favor the formation of high-coloration red intermediate products, thereby significantly improving the sensory quality of the soybean paste color.

In conclusion, microwave puffing (400 W, 90 s) pretreatment significantly enhances the hydrolytic efficiency of proteases and amylases in the fermentation system by altering the structural characteristics of soybean raw materials. It promotes the enrichment of flavor components such as organic acids, amino acid nitrogen, peptides, and reducing sugars, while optimizing the color and quality of the soybean paste. Overall, it improves the fermentation degree and flavor potential of the soybean paste.

### 3.4. Effect of Microwave Puffing Treatment on Umami of Soybean Paste

Previous studies have confirmed that the umami-enhancing effect of small peptides is significantly stronger than that of free amino acids, making them the key component responsible for the core umami flavor of soybean paste [58]. As important umami-enhancing compounds, umami peptides are widely present in soybean paste systems. Compared with other umami-enhancing substances, umami peptides not only possess high umami intensity and a significant umami-enhancing effect but also impart a mellow and rich mouthfeel to the product; they are the primary contributors to the umami quality and full-bodied flavor of soybean paste [59]. To clarify the effects of microwave puffing pretreatment on the yield of small peptides and the umami characteristics of soybean paste during fermentation, this study employed LC-MS/MS technology to systematically analyze the peptide sequences, molecular weight distributions, and amino acid compositions of water extracts from MSP–90 and SP paste fermented for 30 days. Through collision-induced dissociation (CID) or high-energy collision dissociation (HCD) techniques, protonated amide bonds in peptides were cleaved, generating b- and y-type ions in the mass spectrum. These product ions serve as key features for accurately inferring peptide amino acid sequences [60].

**Figure 7:**
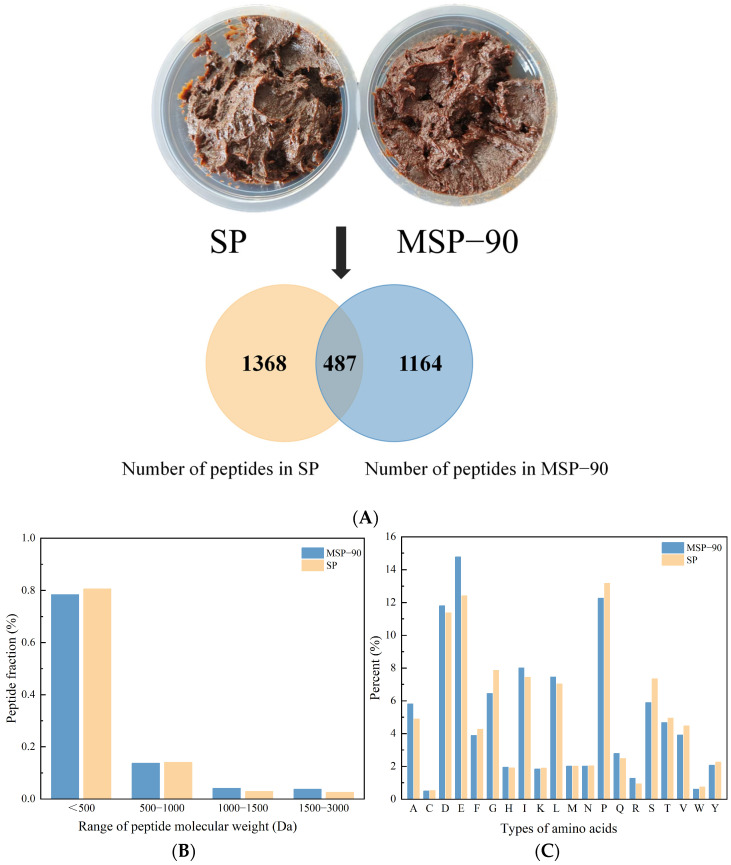
The peptide profile analysis of MSP–90 and SP fermented soybean paste: (**A**) peptide number; (**B**) molecular weight distribution; and (**C**) amino acid distribution of SP and MSP–90 fermented soybean paste.

The results of the peptide profile analysis of the MSP–90 and SP fermented soybean paste extracts after 30 days of fermentation are shown in Figure 7. As shown in Figure 7A, a total of 1164 peptide fragments with molecular weights below 3000 Da were identified in MSP–90, slightly fewer than the 1368 fragments in the control group (SP). Among these, only 487 peptide fragments were common to both groups, indicating that microwave puffing (400 W, 90 s) pretreatment significantly reshaped the peptide profile of the soybean paste. The results of the peptide molecular weight distribution, as shown in Figure 7B, indicate no significant difference in the overall proportion between the MSP–90 and SP groups, and the predominant peptides are concentrated in the 0–500 Da range. Furthermore, since amino acid composition is a key factor determining the umami characteristics of small peptides, this study further analyzed the amino acid composition of the identified peptides, as shown in Figure 7C. It can be observed that, compared to the abundance of other amino acids, the relative abundances of Glu- and Asp-typical umami amino acids were significantly higher in the polypeptides of the MSP–90 and SP soybean paste extractions. Furthermore, the relative abundances of Glu and Asp in the MSP–90 peptides were significantly higher than those in the SP peptides. Mee-Ra et al. [61] suggest that protein hydrolysates rich in bound glutamic acid and with a high proportion of aspartic acid are considered important contributors to the umami flavor of soybean paste. Furthermore, as a typical sweet-tasting amino acid, the proportion of Ala in MSP is also significantly higher than in SP. Hao et al. [62] propose that Ala, as a sweet amino acid, can modulate the sweetness of peptides and enhance umami. At the same time, the relative abundances of typical bitter amino acids, such as Phe, Tyr, Trp, and Val, were significantly lower in SP. These results indicate that microwave puffing causes the rupture of soybean cell walls, while high temperatures denature soybean proteins, disrupting their secondary and tertiary structures. This process breaks intramolecular hydrogen bonds, hydrophobic interactions, and disulfide bonds, transforming the highly compact structure into a more loosely packed one. Consequently, key sites previously hidden within the protein’s internal structure are exposed, providing proteases with more accessible cleavage sites, significantly enhancing protease activity. The abundance of effective cleavage sites enables proteases to perform deep hydrolysis of the substrate proteins with greater specificity, amplifying the proteases’ selective cleavage of umami-rich peptide segments. This shifts the direction of enzymatic hydrolysis from diverse to umami-specific, ultimately achieving the preferential release of peptide segments rich in umami-conferring amino acids.

In summary, microwave puffing (400 W, 90 s) pretreatment significantly reshaped the peptide profile of the soybean paste. Compared to SP, MSP–90 did not increase the total number of peptide segments but significantly increased the proportion of umami amino acids (Glu and Asp) and the sweet amino acid Ala in the peptide segments, while simultaneously reducing the relative abundance of bitter amino acids (Phe, Tyr, Trp, and Val) in the peptide segments. This change in the composition of amino acids in the peptide is the key factor underlying the superior flavor of MSP–90 soybean paste compared to SP soybean paste.

### 3.5. Effect of Microwave Puffing Pretreatment on the Taste of Soybean Paste

**Figure 8:**
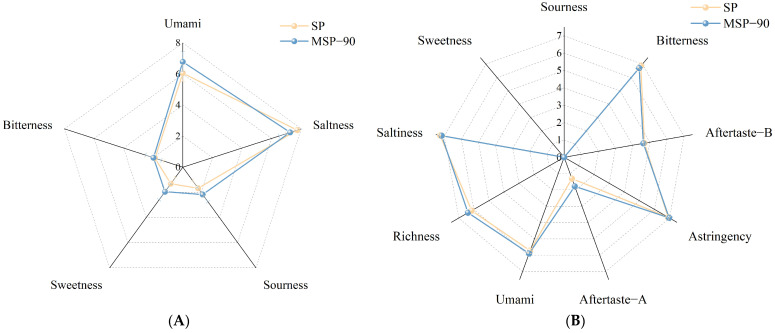

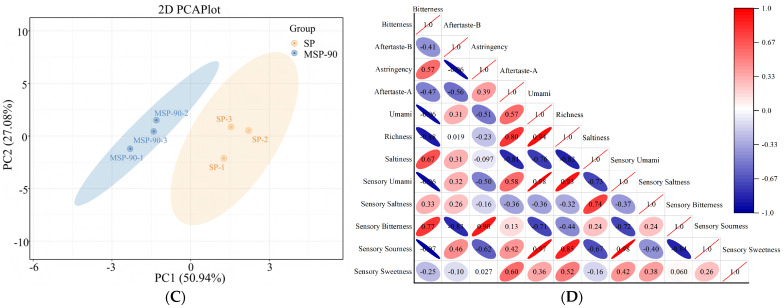
Taste characteristics of MSP–90 and SP fermented soybean paste: (**A**) sensory evaluation; (**B**) electronic tongue analysis; (**C**) principal component analysis score plot; and (**D**) correlation between electronic tongue and sensory evaluation.

Artificial sensory evaluation and electronic tongue analysis were systematically employed to characterize the regulatory effects of microwave puffing pretreatment on the flavor profile of soybean paste [63]. Figure 8A presents a radar chart of the scores for five basic taste attributes (sourness, saltiness, bitterness, sweetness, and umami) of two groups of soybean paste by a professionally trained sensory evaluation panel. The results show that, except for the saltiness score, the scores for umami, sourness, and sweetness of MSP–90 were slightly higher than those of SP, indicating that microwave pretreatment has a potential optimizing effect on the flavor of soybean paste.

Notably, the analysis of the electronic tongue further confirmed the results of the artificial sensory evaluation. As a new technology for rapid detection of taste quality, the electronic tongue can detect taste substances in a way similar to human taste perception, digitize the taste of samples, and has high sensitivity, reliability, and repeatability [64]. It is an effective means for evaluating the taste of soybean paste. To explore the differences in key taste attributes between the MSP–90 and SP groups of soybean paste, we conducted electronic tongue tests on the umami, richness, saltiness, sourness, sweetness, bitterness, aftertaste-b, astringency, and aftertaste-a of the soybean paste. The results are shown in Figure 8B. From the perspective of a single-index taste response, the response values of sourness and sweetness of both groups of soybean paste were 0.00, and no effective signals were detected. This is completely consistent with the flavor characteristics of traditional soybean paste, which is mainly salty and umami, with unremarkable sour and sweet flavors. This phenomenon is closely related to the fermentation and metabolic characteristics of soybean paste. On the one hand, the organic acids produced by microorganisms undergo a neutralization reaction with substances in the fermentation system or are further metabolized, resulting in the free acid concentration in the system being lower than the detection threshold of the electronic tongue. On the other hand, during the fermentation process, polysaccharides such as starch are completely degraded by microorganisms or participate in the Maillard reaction, resulting in an extremely low content of free sugars in the system. Therefore, sourness and sweetness were not detected. It can be seen that the microwave puffing pretreatment did not change the core metabolic pathway of soybean paste fermentation but only regulated the generation of secondary flavor substances. Among the effective taste indicators, there were significant differences between the two groups of soybean paste. The response values of umami and richness of the MSP−90 group were significantly higher than those of the SP group (*p* < 0.05). This is mainly attributed to the modification effect of microwave puffing on soybean protein. The thermomechanical effect of microwaves destroys the high-level structure of proteins, exposes the hydrophobic amino acid residues inside the proteins, and promotes the moderate degradation of proteins, providing more accessible sites for the action of microbial proteases during the fermentation process, accelerating the generation of umami-enhancing peptides and free amino acids (Glu and Asp) [65], and thus enhancing the umami characteristics of soybean paste. At the same time, microwave treatment promotes the formation of some Maillard reaction intermediates, further strengthening the umami synergistic effect of the system and making the richness more persistent. Compared with the SP group, the response values of bitterness, aftertaste-b, and saltiness of the MSP−90 group were significantly lower (*p* < 0.05). This may be because the protein denaturation induced by microwaves may reduce the generation of some bitter peptides or promote the degradation of bitter substances, reducing the intensity of aftertaste-b. At the same time, the generated umami peptides and free glutamic acid further inhibit the perception of bitterness through the taste synergistic effect. In addition, the interaction between umami substances and Na^+^ also weakens the response of the electronic tongue to saltiness, ultimately resulting in a significant decrease in the bitterness, aftertaste-b, and saltiness indicators of the MSP–90 group.

To further elucidate the overall taste profile differences between the SP and MSP–90 groups of soybean paste, principal component analysis (PCA) was applied to the electronic tongue response signals for dimensionality reduction and visual assessment of sample distribution and intergroup separation. As shown in Figure 8C, PC1 and PC2 collectively accounted for 78.02% of the total variance (PC1: 50.94%; PC2: 27.08%), confirming that the PCA model effectively captured the dominant sources of taste variation across samples. Clear separation between the SP and MSP–90 groups along the PC1 axis indicates a statistically meaningful divergence in their global taste response patterns. Concurrently, tight intra-group clustering of biological replicates demonstrates high experimental reproducibility within each treatment. In addition, a Pearson correlation analysis was performed between the effective response values of the electronic tongue and the results of sensory analysis, as shown in Figure 8D. The results show that the umami, richness, and saltiness intensities measured by the electronic tongue were significantly positively correlated with the sensory evaluation scores for umami and saltiness. This finding confirms that the electronic tongue and sensory evaluation exhibit good agreement regarding umami and saltiness. Combining the electronic tongue with sensory evaluation helps yield more reliable research results. It further confirms the significant regulatory effect of microwave puffing pretreatment on the overall taste characteristics of fermented soybean paste.

## 4. Conclusions

In summary, optimal microwave puffing (400 W, 90 s) effectively modulates the conformation of soy protein. The disruption of protein non-covalent bonds disaggregates large proteins into smaller fragments, narrowing the particle size distribution. Hydrophobic groups originally buried within the molecules were exposed, increasing surface hydrophobicity and forming a loose, porous structure, which created favorable conditions for proteolytic hydrolysis during subsequent soybean paste fermentation. In contrast, excessive microwave treatment (>90 s) induces protein molecular re-aggregation, thereby inhibiting enzymatic hydrolysis. Furthermore, microwave puffing (400 W, 90 s) promotes the growth of *Aspergillus oryzae* spores and enhances proteinase activity. Such improvements reduce the mash pH and increase the contents of total acid, amino acid nitrogen, small peptides, and reducing sugars, while endowing fermented soybean paste with a redder and brighter color. Importantly, microwave puffing (400 W, 90 s) reshaped the peptide profile of fermented soybean paste by increasing the proportion of umami amino acids (Glu, Asp) and the sweet amino acid Ala in the peptides, while decreasing the relative abundance of bitter amino acids (Phe, Tyr, Trp, and Val) in the peptides. Sensory evaluation and electronic tongue confirmed that the MSP–90 exhibits superior umami intensity with attenuated bitterness, aftertaste bitterness, and saltiness. PCA further confirms that distinct flavor differences between groups, demonstrating that microwave puffing is a promising pretreatment strategy for regulating the flavor quality of soybean paste.

In conclusion, microwave-induced structural modification of soy protein optimizes the efficiency of protein hydrolysis during fermentation and promotes the release of peptides rich in umami and sweet amino acids. This approach holds significant practical value for enhancing the flavor profile and overall quality of soybean paste. This study is limited by fixed microwave power. At the same time, we speculate that microwave-induced changes in protein structure may alter the cleavage preferences of proteases, thereby shifting the direction of protein degradation and enriching umami-related peptides. Future studies should integrate macroproteomics and enzyme kinetics experiments to thoroughly analyze the interaction between key proteases and their substrates induced by microwave-puffed modified proteins. This will further elucidate the intrinsic relationship between protein structural evolution and enzymatic metabolism and reveal the molecular mechanisms underlying the formation of umami peptides during fermentation. This work provides a theoretical basis for the industrial production of high-quality soybean paste and supports the extended application of microwave modification technology in protein-based fermented food manufacturing.

## Figures and Tables

**Table 1:** Determination of the physicochemical properties of MSP–90 and SP.

Sample	pH	Total Acid (g/100 g)	Amino Acid Nitrogen Content (g/100 g)	Protease Activity (U/g)	Small Peptide (mg/mL)	Reducing Sugar Content (g/100 g)	Moisture Content (%)	L*	a*	b*
MSP–90	4.87 ± 0.02 ^b^	0.73 ± 0.05 ^a^	1.99 ± 0.02 ^a^	300.874 ± 17 ^a^	108.15 ± 0.47 ^a^	3.69 ± 019 ^a^	52.8% ± 0.05 ^b^	29.95 ± 0.08 ^a^	5.53 ± 0.05 ^a^	7.62 ± 0.12 ^b^
SP	5.26 ± 0.03 ^a^	0.69 ± 0.02 ^b^	1.78 ± 0.05 ^b^	195.979 ± 68 ^b^	95.46 ± 0.97 ^b^	3.21 ± 0.10 ^b^	56.5% ± 0.07 ^a^	28.50 ± 0.22 ^b^	4.84 ± 0.17 ^b^	7.80 ± 0.31 ^a^

Values (mean ± SD, *n* = 3) in the same line followed by a different letter are significantly different (*p* < 0.05) according to Duncan’s multiple range test.

## Data Availability

The original contributions presented in this study are included in the article. Further inquiries can be directed to the corresponding authors.
